# Psychiatric Morbidity among a Sample of Orphanage Children in Cairo

**DOI:** 10.1155/2012/141854

**Published:** 2012-12-09

**Authors:** Mohamed A. EL Koumi, Yasser F. Ali, Ehab A. El Banna, Usama M. Youssef, Yasser M. Raya, Aly A. Ismail

**Affiliations:** ^1^Departments of Pediatrics, Faculty of Medicine, Zagazig University, Egypt; ^2^Department of Psychiatry, Faculty of Medicine, Zagazig University, Egypt; ^3^Department of Psychiatry, Faculty of Medicine, Al Azhar University, Egypt

## Abstract

*Objective*. This study identifies the prevalence of emotional and behavioral problems and the associated factors in orphanage children. *Methods*. This cross-sectional study was conducted in three private orphanages in Cairo. Two hundred sixty-five children of ages ranging from 6 to 12 years living in three different orphanages care systems were included in the study. A sociodemographic information form and the Child Behavior Checklist (CBCL) were used. Children were clinically interviewed and psychiatric disorders were identified. Diagnoses were done according to the manual for diagnosis and statistics of mental disorder fourth version (DSMIV). A written formal consent from the director of social solidarity was obtained before inclusion in the study. *Results*. The prevalence of behavioral disturbances was 64.53% among those in institutional care and the most prominent psychiatric disorders were nocturnal enuresis (23.3%), attention deficit hyperkinetic disorder (ADHD) (19.62%), oppositional defiant disorder (17.36%). Age at first admission, causes of receiving institutional care, and moves 2 or more times between institutions were significantly associated with an increased risk of behavioral and emotional problems. *Conclusion*. Our study showed that children living in institutions are prone to suffer from psychiatric disorders. Stability of the caregiver acts as a protective variable.

## 1. Introduction

Childhood is a developmental stage in which the importance of reciprocal emotional bonding between a child and his/her caregivers, for healthy physical, psychological, and social development has been known for centuries [1]. Orphans in group homes or institutions take more risks, have more threats to achievement, and have poorer peer influences [2]. Almost no systematic studies have been carried out during the past five decades about orphanages largely because nearly all orphanages in industrial nations have been replaced by adoption and foster care [3]. This solution, in Third World Countries, is unacceptable either religiously in some countries as Egypt, or has been considered an unrealistic solution in other countries as in Africa [3]. Although orphanages can provide a secure and positive alternative to abusive and unsafe family or community environments, they cannot provide individualized and family nurturing [4]. Research findings indicate that children in institutional care have more behavioral problems, such as aggressive behavior and have higher levels of depression and anxiety, compared to children that are reared in a family environment [5–7]. Several studies have shown that institutional care has negative effects, especially on young children; however, only a few studies have investigated the prevalence rate of problems among these children, the extent of care service needs, and risk and protective factors by collecting data from multiple informants. Early intervention immediately following institutional care placement is recommended [8–12].

## 2. Methods

This cross-sectional epidemiological study recruited 265 children (190 males and 75 females) of ages ranging from 6 to 12 years (X ± SD: 3.35 ± 0.9 years) residing in three orphanages in Cairo and who had lived with their caregivers for at least a month during the year 2011 through 2012. Psychiatric analysis was performed by 3 experienced psychiatrists. Excluded from the survey; children with visual and/or auditory deficits, children younger than 6 years, and those with chronic medical illnesses. Children from orphanage I were 81, orphanage II were 147, and orphanage III were 37. Children were divided into three groups according to age.

*Group 1*: included 108 children of ages ranging from 6 to 8 years. *Group 2*: included 70 children of ages ranging from 8 to 10 years. *Group 3*: included 87 children of ages ranging from 10 to12 years. All children were subjected to the following.

1. *Sociodemographic Information Form*: this form collected data about risk and protective factors regarding children and their families. Based on the information in each child's file, a social service specialist or a psychologist completed the form. Predictive factors, such as current age, age at first admission, history for admission, gender, place of residence prior to institutional care, the frequency of moving from one institution to another and death of mother/father were recorded.
2. *Child Behavior Checklist (CBCL/6–18)*: the CBCL is designed to obtain parents'/caregivers' reports of their children's problems. The CBCL was translated to Arabic and its reliability and validity has been established The test retest reliability was 0.84 for total problems and 0.88 for internal consistency. In the validity study, confirmatory factor analysis was conducted and 99% of the items were found to be significantly, positive, and sufficiently measure what they were designed to measure (*P* < 0.01) [13]. The CBCL includes items for rating competencies and 113 items for behavioral and emotional problems. Items are rated on a 3-pointscale as 0 = not true, 1 = sometimes true, and 2 = often true, based on the preceding 6 months. The following syndromes are scored from the CBCL: Internalizing group (anxious/depressed, withdrawn/depressed, or somatic complaints), externalizing group (rule breaking behavior, or aggressive behavior), social problems, thought problems, and attention problems. [14–16].
3. *Semistructured Psychiatric Interview according to DSM IV*. To detect presence of psychiatric disorders by a qualified psychiatrist.

## 3. Statistical Analysis 

Data were presented as mean ± standard deviation (X ± SD) or percentage (%). The means of two groups were compared using student's *t* test. Data were tabulated and statistically analyzed with the statistical package for social sciences (SPPS) version 14 software. *P* values less than 0.05 were considered significant.

## 4. Results

Two hundred and seventy five children were identified as being eligible to participate in the study. Of these, caregivers of 10 children declined to participate. This left 265 participating children who were residing with 81 caregivers. More than half the children 71.7% had been in their current placement for less than 2 years; 74.71% of children had a female caregiver 64.52% had a stable caregiver for more than 6 months (Table 1).

The range of total score was 31–69 (mean = 43.78); the highest scores were on externalizing, internalizing, and aggressive score and the least was somatic score (Table 2).

The completed Child Behavior Checklist (CBCL) showed 11 Ratings on the questionnaire summarized as a total behavior problems score comprising all items on the checklist. Children had total behavior problems represent 61,13% of the studied group. The externalizing factors were higher than the internalizing ones (60% versus 58.86%); the most predominant problem was attention (43,77%) whereas somatic problem was the least (18.11%) (Table 3).

The psychiatric diagnosis of the whole sample (No. 265) as shown in (Table 4); enuresis (23.03%) was the commonest diagnosis on axis I and mental retardation (6%) on axis II. While on axis I attention deficit hyperactivity disorder (19.62%), oppositional defiant disorder (17.36%), sleep disorders (10.75%) followed by conduct disorder (9.81%) depression disorder (7.17%), separation anxiety disorder (7.17%), lastly no diagnosis on Axis I (35.47%), less than (1%) tics, dysthymia, and schizophrenia.

Statistical correlation is shown between different psychiatric morbidity diagnoses and other variables including age differences, sex of caregiver, stability of caregiver reason for institutionalization, moves between institutions, and length of institutionalization (Tables 5(a) and 5(b)).

## 5. Discussion

Lately, an increase in the number of orphanages in many of the Egyptian governorates was noticed as a response of the orientation that encourages the care for the orphans. Many child care professionals are still believing that orphanages are bad for children and they will fail to have a normal development both socially and psychologically [17]. Orphanage children are uniquely vulnerable to many psychosocial hazards of institutional care [18]. Fisher et al. [12], Vorriat et al. [19], Roy et al. [20], and MacLean [21], compared children in institutional care and children living with their families andere found that institutional care had negative effect on the emotional and behavioral development of children aged 6–12 years. This study of psychiatric and behavioral problems in orphans has revealed that the prevalence rate of total behavior problems was (61%), compared to other studies that used CBCL which detected the prevalence rate of behavior problems was nearly 20%–78% [22], and 33% [17]. In our study the main increase in score of CBCL was in the externalizing factors (hyperactive, aggressive, and delinquent) 60.1%. This denotes the overt aggressive and rebellious attitude of these orphans towards society as they grow up and become more aware of their real condition in the society. They live in conflict that they resolve by indulging in violence. Another cause of this was suggested to be the separation of a child from his/her parents at early ages and the related attachment problems that lead to externalization problems [23]. Cantone et a1. [24]. stated that the internalizing and externalizing factors represent a distinction between fearful inhibited over controlled behavior (internalizing) and aggressive antisocial uncontrolled behavior (externalizing). Other internalizing factors as depressed factor has shown a highly significant increase explained by being deprived of families and lacking the essential psychological support at this early stage of life. The change in total scores of CBCL in the followup was of no statistical significance. In our study, there was statistically significant increase in separation anxiety disorder in children with the lowest age. Offord et al. [25] and Miller et al. [26] had found that frequency and severity of antisocial behavior varied directly with age of adoption. According to the reports of multiple informants, institutionalization at younger ages, the conditions of previous living in another institutions, two or more moves between institutions, and recurrent physical illness increased the risk of emotional and behavioral problems [12, 27, 28]. Caregiver quality was the most important predictive factor for behavioral problems, which is compatible with previous studies [29]. One of the leading factors that ensure the mental health of children is the quality of the relationship with a caregiver, which is marked by confidence, support, continuity, and warmth. For this reason, caregiver training is identified as one of the most important preventive interventions. 

The most prevalent diagnosis was enuresis (23.03%); a finding very close to that was found by Abolmagd (22.9%) in orphans compared to (6.7%) of the school children [30]. On the other hand, El-Ray [31] has found that (41.1%) of orphans were diagnosed as enuretic. This is explained psycho analytically according to Garfinkel [32] as an expression of passive aggressive behavior, depression, and inability to resolve or contain high levels of anxiety. Meanwhile, Essen and Peckham [33] added that socioeconomic status and stressful life events characteristic of family dysfunction are also more frequently associated with enuresis. Some Egyptian surveys estimated nocturnal enuresis in Egyptian children by 1.9%, out of them, disturbances of home atmosphere was found in (61%) of enuresis children [34]. It is noticed that children in orphanages demonstrated aggression either passively through enuresis or explicitly as an overt aggressive behavior. Sleep disorders (10.57%) were present of all its types and it was noticed that one cause for it may be the hard program put for the children [35]. Current study demonstrated speech disorders affecting 1.5% of the sample possibly due to anxieties or lack of care in orphanages. Okasha et al. [36], showed that stammers have delayed milestones, aggression, instability and shyness. On the other hand,separation anxiety disorder and depression disorder were found in 7.17% and 7.17% of cases, respectively, which may be explained by various variables including all the children had lost one of their parent before the age of 11 years, all were from lower socioeconomic class which are known risk factors for the development of depression [37]. However, These rates are somewhat lower than those found by Fawzy and Fouad (21% and 58.5% resp.,) and far lower than those found in other countries, which is attributed possibly, in Egypt, to the sympathy, social support, and religious backgrounds [38]. In Axis II, mental retardation was present in 6% of our cases, a figure which is nearer to that found by Abolmagd (12.1%) of orphan children versus (2.7%) of the control group [30] but far less than that found by El-Ray, who found that (24.4%) of orphans were mentally retarded [31]. This can be attributed to many possible factors as reported by Grantham-McGregor and Ani [39] including undernutrition, border line intelligent mother, physical or sexual abuse, and/or low previous socioeconomic standards.

## 6. Conclusion

In Egypt, orphanage harbors either true orphans or foundling abandoned by mothers after illegitimate pregnancies. This study showed that children living in orphanages are more prone to suffer from various psychiatric disorders and the stability of caregiver acts as a protective variable. Given the high prevalence of psychiatric morbidities in such institutions and to avoid its hazardous effect on the community, we recommend proper supervision of the orphanages by the supervising authorities, regular training courses for the caregivers to help improving their children caring skills, and psychiatric surveillance for the orphanages must be available and continuous for early detection and treatment of psychiatric disorders. Finally, More studies on orphanages should be carried out including bigger number with longer period of followup in other Egyptian governorates.

## Figures and Tables

**Table 1:** Sociodemographic characteristics of the studied children.

	Number (*n*)	Percent (%)
Orphanage		
I	81	30.56
II	147	59.47
III	37	13.96
Gender		
Male	190	71.7
Female	75	28.3
Age (years)		
6-7	108	40.75
8-9	70	26.41
10–12	87	32.82
Reasons for institutionalization		
Abandonment	146	55.09
Orphanage	64	24.15
Disturbed family	55	20.75
Moves between institution		
1	150	56.6
2	97	36.6
3	18	6.8
Gender of caregivers		
Male	67	25.3
Female	198	74.7
Stability of caregivers		
Stable	171	64.5
Unstable	94	35.5
Length of institution		
Less than or equal to 2 years	190	71.7
More than 2 years	75	28.3

**Table 2:** Descriptive statistics for the child behavioral checklist.

	Minimum	Maximum	Mean	Std. deviation
Anxiety subscale	35	60	45.31	6.52
Withdrawal subscale	34	55	45.789	7.778
Thought problem	30	35	30.99	5.50
Somatic complains	30	33	31.15	6.60
Social problems	30	35	32.07	6.57
Attention	30	36	31.04	11.9
Aggression	31	69	48.98	9.87
Delinquent	30	61	40.18	7.41
Internalizing subscale	43	69	56.47	7.77
Externalizing subscale	31	69	49.356	8.60

**Table 3:** Children identified by the caregivers using CBCL to have behavioral problems.

Problem	Number (*n*)	Percent (%)
Total behavior problems	162	61.13
Externalizing problems	159	60
Internalizing problems	156	58.86
Attention problems	116	43.77
Social problems	110	41.5
Delinquent behavior	108	40.75
Aggressive behavior	101	38.11
Thought problems	96	36.3
Withdrawn	61	23
Anxious/depressed	53	20
Somatic complaints	48	18.11

**Table 4:** Psychiatric diagnoses of the orphans according to DSM IV.

Diagnoses	Number (*n*)	Percent (%)
At least one diagnosis	171	64.53
More than one diagnosis	95	35.85
Enuresis	61	23.03
Nocturnal	52	19.62
Diurnal	2	0.75
Both	7	2.64
ADHD	52	19.62
Hyperactivity	36	13.58
Attention deficit	10	3.77
Both	6	2.26
Separation anxiety disorder	19	7.17
Oppositional defiant disorder	46	17.36
Speech disorders	4	1.51
Sleep disorders	28	10.57
Conduct disorder	26	9.81
Depression	19	7.17
Tics	2	0.78
Learning disorders	6	2.26
Dysthymia	2	0.78
Schizophrenia	1	0.38
Mental retardation	16	6

**Table:** (a)

Variables	Enuresis		ADHD		Separation anxiety disorder		Oppositional defiant disorder		Speech disorders		Sleep disorders	
	*n*	*P*	*n*	*P*	*n*	*P*	*n*	*P*	*n*	*P*	*n*	*P*
Gender												
Male	45	0.108	37	0.732	13	0.388	32	0.786	4	0.286	22	0.623
Female	16		15		6		14		0		6	
Age (years)												
6–8	33		18		12		18		2		14	
8–10	12	0.0368	19	0.055	1	0.0001*	21	0.641	1	0.844	6	0.399
10–12	16		15		6		7		1		6	
Reason of institutionalization												
Abandonment	30		30		14		26		3		13	
Orphanage	14	0.732	13	0.5	2	0.0001*	14	0.348	1	0.577	1	0.0001*
Disturbed family integrity	17		9		3		6		0		14	
Moves between institutions												
1	27		39		8		24		4		12	
2	28	0.546	13	0.0857	9	0.355	15	0.331	0	0.001*	10	0.449
3	6		0		2		7		0		6	
Sex of caregiver												
Male	13	0.202	15	0.936	7	0.584	4	0.941	1	0.0001*	5	0.601
Female	84		37		12		42		3		23	
Stability of caregiver												
Stable >6 months	42	0.972	37	0.935	15	0.453	32	0.914	1	0.0001*	19	0.593
Unstable	20		15		4		14		3		9	
Length of institutionalization												
Less than 2 years	37	0.357	43	0.793	15	0.366	37	0.968	1	0.017*	22	0.623
More than 2 years	24		9		4		9		3		6	

*P* < 0.05 Significant, *Significant, *n*: number, ADHD: attention deficit hyperkinetic disorder.

**Table:** (b)

Variables	Conduct disorders		Depression		Tics		Learning disorders		Dysthymia		Schizophrenia	
	*n*	*P*	*n*	*P*	*n*	*P*	*n*	*P*	*n*	*P*	*n*	*P*
Gender												
Male	38	0.907	16	0.360	1	0.001*	4	0.004*	1	0.001*	0	0.0001*
Female	8		3		1		2		1		1	
Age (years)												
6–8	11		10		0		3		0		0	
8–10	12	0.279	5	0.923	1	0.844	0	0.623	2	0.0001*	1	0.661
10–12	3		4		1		3		0		0	
Reason for institutionalization												
Abandonment	10		6		1		3		2		1	
Orphanage	11	0.620	8	0.290	1	0.619	2	0.086	0	0.094	0	0.391
Disturbed family integrity	5		5		0		1		0		0	
Moves between institutions												
1	16		8		0		1		1		0	
2	6	0.492	7	0.357	2	0.279	2	0.216	0	0.546	1	0.923
3	4		4		0		1		0		0	
Sex of caregiver												
Male	3	0.538	7	0.584	0	0.0001*	1	0.0001*	2	0.125	0	0.215
Female	23		12		2		5		1		1	
Stability of caregiver												
Stable >6 months	17	0.507	10	0.575	2	0.123	3	0.018*	0	0.003*	0	0.235
Unstable	9		9		0		3		2		1	
Length of institutionalization												
Less than 2 years	19	0.489	13	0.387	0	0.0001*	4	0.043*	1	0.001*	0	0.007*
More than 2 years	7		6		2		2		1		1	

*P* < 0.05 significant, *Statistically significant.

## References

[1] Erol N, Simsek Z, Münir K (2010). Mental health of adolescents reared in institutional care in Turkey: challenges and hope in the twenty-first century. *European Child and Adolescent Psychiatry*.

[2] Altshuler SJ, Poertner J (2002). The child health and illness profile-adolescent edition: assessing well-being in group homes or institutions. *Child Welfare*.

[3] Wolff PH, Fesseha G (1999). The orphans of Eritrea: a five-year follow-up study. *Journal of Child Psychology and Psychiatry and Allied Disciplines*.

[4] Garvin MC, Tarullo AR, Van Ryzin M, Gunnar MR (2012). Postadoption parenting and socioemotional development in postinstitutionalized children. *Development and Psychopathology*.

[5] Trout AL, Casey K, Chmelka MB, DeSalvo C, Reid R, Epstein MH (2009). Overlooked: children with disabilities in residential care. *Child Welfare*.

[6] Vorria P, Papaligoura Z, Dunn J (2003). Early experiences and attachment relationships of Greek infants raised in residential group care. *Journal of Child Psychology and Psychiatry and Allied Disciplines*.

[7] Johnson R, Browne K, Hamilton-Giachritsis C (2006). Young children in institutional care at risk of harm. *Trauma, Violence, and Abuse*.

[8] Leite LC, Schmid PC (2004). Institutionalization and psychological suffering: notes on the mental health of institutionalized adolescents in Brazil. *Transcultural Psychiatry*.

[9] Tizard B, Hodges J (1978). The effect of early institutional rearing on the development of eight year old children. *Journal of Child Psychology and Psychiatry and Allied Disciplines*.

[10] McCann JB, James A, Wilson S, Dunn G (1996). Prevalence of psychiatric disorders in young people in the care system. *British Medical Journal*.

[11] Ahmad A, Qahar J, Siddiq A (2005). A 2-year follow-up of orphan’s competence, socioemotional problems and post-traumatic stress symptoms in traditional foster care and orphanages in Iraqi Kurdistan. *Child*.

[12] Fisher L, Ames EW, Chisholm K, Savoie L (1997). Problems reported by parents of romanian orphans adopted to British Columbia. *International Journal of Behavioral Development*.

[13] El Defrawy MH, Abd El Wahab AM, Atef A, Abd El Hady M (1995). *Arabic Translation of Achenbach's Child Behavior Checklist*.

[14] Palen LA, Coatsworth JD (2007). Activity-based identity experiences and their relations to problem behavior and psychological well-being in adolescence. *Journal of Adolescence*.

[15] Tan TX, Dedrick RF, Marfo K (2007). Factor structure and clinical implications of Child Behavior Checklist/1.5-5 ratings in a sample of girls adopted from China. *Journal of Pediatric Psychology*.

[16] Biederman J, Monuteaux MC, Kendrick E, Klein KL, Faraone SV (2005). The CBCL as a screen for psychiatric comorbidity in paediatric patients with ADHD. *Archives of Disease in Childhood*.

[17] Lassi ZS, Mahmud S, Syed EU, Janjua NZ (2011). Behavioral problems among children living in orphanage facilities of Karachi, Pakistan: comparison of children in an SOS Village with those in conventional orphanages. *Social Psychiatry and Psychiatric Epidemiology*.

[18] Frank DA, Klass PE, Earls F, Eisenberg L (1996). Infants and young children in orphanages: one view from pediatrics and child psychiatry. *Pediatrics*.

[19] Vorriat P, Wolkind S, Rutter M, Pickles A, Hobsbaum A (1998). A comparative study of Greek children in long-term residential group care and in two-parent families: I. Social, emotional, and behavioural differences. *Journal of Child Psychology and Psychiatry and Allied Disciplines*.

[20] Roy P, Rutter M, Pickles A (2000). Institutional care: risk from family background or pattern of rearing?. *Journal of Child Psychology and Psychiatry and Allied Disciplines*.

[21] Maclean K (2003). The impact of institutionalization on child development. *Development and Psychopathology*.

[22] Burns BJ, Phillips SD, Wagner HR (2004). Mental health need and access to mental health services by youths involved with child welfare: a national survey. *Journal of the American Academy of Child and Adolescent Psychiatry*.

[23] Tehrani-Doost M, Shahrivar Z, Pakbaz B, Rezaie A, Ahmadi F (2011). Normative data and psychometric properties of the child behavior checklist and teacher rating form in an Iranian community sample. *Iranian Journal of Pediatrics*.

[24] Cantone D, Sperandeo R, Maldonato MN, Cozzolino P, Perris F (2012). Dissociative phenomena in a sample of outpatients. *Rivista di Psichiatria*.

[25] Offord DR, Aponte JF, Cross LA (1969). Presenting symptomatology of adopted children. *Archives of General Psychiatry*.

[26] Miller L, Chan W, Comfort K, Tirella L (2005). Health of children adopted from Guatemala: comparison of orphanage and foster care. *Pediatrics*.

[27] Beckett C, Maughan B, Rutter M (2006). Do the effects of early severe deprivation on cognition persist into early adolescence? Findings from the English and Romanian adoptees study. *Child Development*.

[28] Miller LC, Tseng B, Tirella LG, Chan W, Feig E (2008). Health of children adopted from Ethiopia. *Maternal and Child Health Journal*.

[29] Zeanah CH (2000). Disturbances of attachment in young children adopted from institutions. *Journal of Developmental and Behavioral Pediatrics*.

[30] Abolmagd S (2000). Psychiatric and emotional disorders in a sample of Egyptian orphanage resident children. *Egyptian Journal of Psychiatry*.

[31] El-Ray LA (1999). Psychiatric and behavioral disorders in a sample of Egyptian orphanage resident children: a preliminary study. *Egyptian Journal of Psychiatry*.

[32] Garfinkel BD, Garfinkel BD,  Carlson GA, Weller EB (1990). The elimination disorders. *Psychiatric Disorders in Children and Adolescents*.

[33] Essen J, Peckham C (1976). Nocturnal enuresis in childhood. *Developmental Medicine and Child Neurology*.

[34] Okasha A (2004). Focus on psychiatry in Egypt. *British Journal of Psychiatry*.

[35] Humphrey JA (1974). A study of the etiology of sociopathic behavior. *Disease of the Nervous System*.

[36] Okasha A, Bishry Z, Kamel M, Hassan AH (1974). Psychosocial study of stammering in Egyptian children. *British Journal of Psychiatry*.

[37] Karadağ Çaman Ö, Özcebe H (2011). Adolescents living in orphanages in Ankara: psychological symptoms, level of physical activity, and associated factors. *Turk Psikiyatri Dergisi*.

[38] Fawzy N, Fouad A (2010). Psychosocial and developmental status of orphanage children: epidemiological study. *Current Psychiatry*.

[39] Grantham-McGregor SM, Ani CC (1999). The role of micronutrients in psychomotor and cognitive development. *British Medical Bulletin*.

